# Prevalence, antibiotic sensitivity profile, and phylogenetic analysis of *Escherichia coli* isolated from raw dromedary camel milk in Matrouh Governorate, Egypt

**DOI:** 10.5455/javar.2022.i578

**Published:** 2022-03-13

**Authors:** Elham Saeed, Amr Abd El-Moamen Amer, Hani Gamal Keshta, Elsayed E. Hafez, Rania M. S. Sultan, Eman Khalifa

**Affiliations:** 1 Department of Food Hygiene, Faculty of Veterinary Medicine, Matrouh University, Mersa Matrouh, Egypt; 2 Department of Food Hygiene, Faculty of Veterinary Medicine, Alexandria University, Alexandria, Egypt; 3 Department of Animal Medicine, Faculty of Veterinary Medicine, Matrouh University, Mersa Matrouh, Egypt; 4 Department of Plant Protection and Biomolecular Diagnosis, ALCRI, City of Scientific Research and Technological Applications Alexandria, Alexandria, Egypt; 5 Department of Biological Sciences, Faculty of Science, King Abdulaziz University, Jeddah, Saudi Arabia; 6 Department of Microbiology, Faculty of Veterinary Medicine, Matrouh University, Mersa Matrouh, Egypt

**Keywords:** *Escherichia coli*, Matrouh Governorate, *phoA* gene, raw dromedary camel milk, sequencing

## Abstract

**Objective:**

Most people in Matrouh Governorate consume camel milk as a treatment for many diseases in a raw state to obtain nutritive value. Raw dromedary camel milk can be contaminated by *Escherichia coli *through fecal matter at any point of milk handling; therefore, it may lose its value and safety specifications. This survey aimed to estimate the incidence of *E. coli *in fresh camel milk.

**Materials and Methods:**

100 fresh camel milk samples (50 from markets and 50 from farms) were randomly collected from different districts in Matrouh Governorate, Egypt, over 4 months for the detection of *E. coli* incidence through conventional bacterial isolation, molecular investigation, and gene sequencing.

**Results:**

The prevalence rates of *E. coli *in the examined market and farm raw camel milk based on conventional methods were 24% and 8%, respectively, while those by molecular identification using *phoA* as an *E. coli *determinate gene were 4% and 6%, respectively. Moreover, *E. coli phoA* gene phylogenetic analysis revealed high sequence similarity to *E. coli* strain CP033158.1 in India and *E. coli* strain CP047594.1 in China. Antibiotic sensitivity of *E. coli* isolates showed high susceptibility to norfloxacin (10 µg) and cefoperazone (75 µg). On the other hand, high resistance was found in rifamycin (30 µg) and cefoxitin (30 µg).

**Conclusion:**

The results indicate that market camel milk is more contaminated than the farms’ own. Additionally, antibiotic resistance is increasing due to antibiotic abuse.

## Introduction

Raw dromedary camel milk is an essential factor in the nomadic diet for its medicinal properties in relation to many diseases and its richness in vitamins, minerals, antimicrobial factors, and antioxidants compared with other animal species [1]. 

Microbial contamination of raw camel milk may have multiple sources, such as the udder, utensils, droplets, cleaning water, dairymen, and dust. Furthermore, its nutritional value, which is good for microbial growth, depends on how long it has been stored [2].

Escherichia coli is a bacteria that is frequently found in human bowels and warm-blooded animals. Many E. coli strains are commensal. Nevertheless, certain strains, such as Shiga toxin-E. coli (STEC), may cause food poisoning, while others cause urinary and respiratory infections and other diseases [3].It is passed to humans through contaminated food consumption, such as raw milk and raw food products [4].

STEC causes gastrointestinal disorders, including non-bloody or bloody diarrhea, and hemolytic uremic syndrome [5]. The occurrence rates of *E. coli *in raw dromedary camel milk in different countries were 8.1% of 24 samples in Harar and Dire Dawa, Eastern Ethiopia; 25.71% of 35 samples in Matrouh Governorate, Egypt; 7.44% of 215 samples from different regions of southern Iraq, but not detected in Giza, Egypt; and 8.5% of 104 samples from Garissa County, Kenya [6–10].

Food intoxication cannot occur in the absence of *stx*. E. colineeds the eae*A* gene to attach, colonize, and then release the toxin, so eae*A* is a significant virulence factor for E. coli pathogenesis and identification, which is responsible for the adherence of E. coli to the gut wall [11].

E. coli antimicrobial resistance has spread around the world, and its susceptibility forms show a lot of geographic variations and differences in people and the environment [12]. Many incidents of antibiotic resistance have been determined in microbes isolated from mastitic milk, such as E. coli, which is considered a warning to human and animal health nowadays [13]. 

The Center for Disease Control and Prevention [14] manages a nationwide network of sequences that are used to identify possible outbreaks. Sequencing provides investigators with data about the food poisoning bacteria. If the isolated bacteria from infected patients were genetically related, this would indicate that patients were infected by the same causative agent.

Sequencing is highly significant in the formation of the global database for foodborne pathogens. This is alarming because it is used to identify unknown genomes and sources of infection in multiyear and multistate outbreaks [15]. 

The current investigation is designed to assess the incidence of E. coli in raw dromedary camel milk gathered from different districts in Matrouh Governorate, Egypt, and screen for the possible presence of its determined gene, virulence genes (which are confirmed by gene sequencing and phylogenetic analysis), and antibiotic-resistant pattern.

## Materials and Methods

### Ethical approval

This study has prior approval from the animal care and use committee institution, Alexandria Uni. (ALEXU-IACUC) member of ICLAS. No. of agreement: AU 005 2019-07-15 MS (1) 02.

### Sample collection

One hundred samples of raw camel milk were randomly gathered from Matrouh Governorate from various markets and farms (50 samples from each) in Siwa, Salloum, Almtani (Dardouma area), and Sidi Barani in the Matrouh desert regions in four consecutive months. Each sample (250 ml) was collected from markets as they were sold in their retail containers and from farms in sterile falcon tubes. The samples were transported to the laboratory of microbiology in a cool box at 4°C ± 1°C within 2–4 h. Each sample of milk was perfectly mixed before being subjected to bacteriological evaluation for E. coli.

### Conventional identification of E. coli

Inoculation and incubation of the selective enrichment medium (lauryl sulfate broth) were carried out as described previously [16]. In brief, 1 ml of raw camel milk was added to each lauryl sulfate broth tube (each tube contains 6 ml lauryl sulfate broth and is supplied with overturned Durham tubes). Then, it was stored at 37°C ± 1°C/24 ± 2 h. The tubes were observed for opacity, cloudiness, and any visible gas; negative tubes were incubated for up to 48 ± 2 h.

Isolation of E. coli on eosin–methylene blue (EMB) agar [17]. A loopful of positive lauryl sulfate broth tube was streaked onto a pre-dried surface of EMB agar medium; then, the petri dishes were stored at 35°C ± 0.5°C/18–24 h. Ideal colonies of E. coli on EMB medium are flat colonies with a dark center, with or without metallic green shine.

### Molecular identification using conventional polymerase chain reaction (PCR)

Molecular identification was conducted in the Lab. for Veterinary Quality Control on Poultry Production, Animal Health Research Institute (AHRI), Giza, Egypt. DNA extraction was performed using the QIAamp DNA Mini Kit (catalog no. 51304) in accordance with the pamphlets. The PCR procedures of each primer pair were conducted according to their parallel reference in Table 1. The products of PCR were subjected to gel electrophoresis [18]and then transferred into a UV cabinet. The gel was pictured using a gel recording system (Alpha Innotech),and the records were examined using software.

### Sequencing

Sequencing was conducted at Elim Biopharmaceuticals, USA. An extracted conventional PCR product was sequenced in the forward and reverse directions on an Applied Biosystems 3130 Automated DNA Sequencer (ABI 3130 USA)using a ready reaction Big Dye Terminator V3.1 cycle sequencing kit (Perkin-Elmer/Applied Biosystems, Foster City, CA)(Cat. No. 4336817).BLAST^®^ analysis [22] was used to create sequence characters for GenBank accessions. Sequence results were conducted corresponding to the guides. The results of nucleotide sequencing were submitted to GenBank via Bankit (GenBank n.d.). The sequences were accepted and received accession numbers.

**Table 1.** Sequences of primers used in conventional PCR.

Objective bacteria	Objective gene segment	Oligonucleotide sequence (5′→3′)	Band (bp)	Reference
*E. coli*	*phoA*	5′ CGATTCTGGAAATGGCAAAAG 3′	720	[19]
		3′ CGTGATCAGCGGTGACTATGAC 5′		
	*stx*1	5′ ACACTGGATGATCTCAGTGG 3′	614	[20]
		3′ CTGAATCCCCCTCCATTATG 5′		
	*stx*2	5′ CCATGACAACGGACAGCAGTT 3′	779	
		3′ CCTGTCAACTGAGCAGCACTTTG 5′		
	*eaeA*	5′ ATGCTTAGTGCTGGTTTAGG 3′	248	[21]
		3′ GCCTTCATCATTTCGCTTTC 5′		

**Table 2.** Incidence of *E. coli *isolated from assessed raw dromedary camel milk samples.

Source	No. of examined samples	Conventional methods		Molecular identification	
		No.	%	No.	%
Market milk	50	12	24	2	4
Farm milk	50	4	8	3	6

### Phylogenetic analysis

Phylogenetic analysis was conducted using MEGA X [23] by comparing the resultant sequences with those available in GenBank. The phylogenetic tree was built according to UPGMA.

### Antibiotic susceptibility testing

Antibiotic sensitivity testing of isolates was carried out by the disk diffusion technique [24].The isolates were exposed to sensitivity tests against norfloxacin (10 µg), cefoperazone (75 µg), pefloxacin (5 µg), ciprofloxacin (5 µg), nitrofurantoin (300 µg), tobramycin (10 µg), rifamycin (30 µg), cefoxitin (30 µg), rifampicin (5 µg), streptomycin (10 µg), neomycin (30 µg), chloramphenicol (30 µg), ofloxacin (5 µg), levofloxacin (5 µg), piperacillin (100 µg), erythromycin (15 µg), and novobiocin (30 µg), which were used in the treatment of most mastitis cases in the city. The areas of complete inhibition were calculated and explained after incubation at 35°C ± 2°C for 24 h.

Multiple antibiotic resistance (MAR) was determined for all isolates through the formula MAR = *a*/*b*, where *a* corresponds to the sum of antibiotics to which isolates were resistant and *b* signifies the total number of antibiotics that were used for sensitivity [25].

## Results and Discussion

*Escherichia coli *existence in raw dromedary camel milk is a threat to human health. The results recorded in Table2 show that *E. coli *could be detected in 24% (12/50) and 8% (4/50) of the assessed market and farm raw camel milk samples, respectively, using conventional biochemical methods. Higher results (31.5%) were reported by [26].

**Figure 1.**
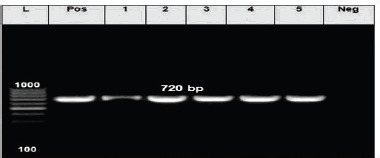
Electrophoretic gel imaging of PCR for *E. coli* *phoA* gene (720-bp). Lane L, DNA (100 bp plus ladder); Lane Pos, control positive strain (*E. coli* ATCC 25922); Lane Neg, control negative strain*; Lanes 1, 2, 3, 4, and 5, +ve strains; Lanes 1 and 2, market raw camel milk isolates; Lanes 3–5, farm raw camel milk isolates*.

The isolated *E. coli *was screened for *phoA* gene using molecular identification. As shown in Table 2 and Figure 1, the prevalence rates were 4% (2/50) in markets and 6% (3/50) in farm. All positive samples did not respond to the Egyptian standard [27],which stipulated that raw milk should be free from *E. coli.*

The market raw camel milk samples were contaminated more than the farms’ own, which indicates that the hazards occurred during filling and transportation through polluted containers and poor storage temperatures [28]. Ruminants are the main reservoirs of STEC. Milk is contaminated with it through mastitis, fecal matter, or contaminated milking utensils [29].

The subsistence of E. coli in raw camel milk is because of fecal contamination by either direct or indirect methods such as poor sanitation during handling, far markets, and lack of refrigerators, which lead to a high bacterial load in the market samples [30]. 

Raw camel consumption is usually followed by diarrheagenic E. coli outbreaks attributable to rough handling procedures. Additionally, a high incidence of pathogenic E. coli strains in fresh milk is detected in many countries all over the world [31].

**Figure 2.**
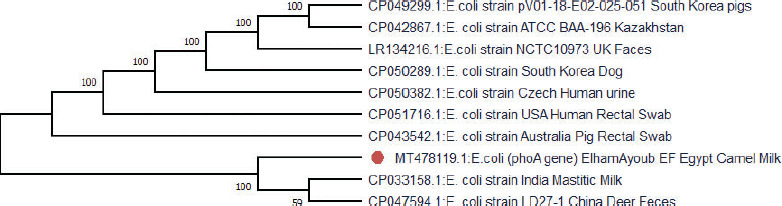
Phylogenetic tree for forward *phoA* gene sequence of *E. coli *compared with other *E. coli *strains from different countries and sources listed in GenBank by UPGMA test.

Figure 2 shows that the phylogenetic analysis of the forward pho*A* sequence of E. coli, which was isolated from raw dromedary camel milk (MT478119), showed high sequence similarity to E. coli strain CP033158.1 in India that were isolated from mastitic milk and E. coli strain CP047594.1 in China that were isolated from deer feces by 59%. This similarity explains the various mechanisms of antibiotic resistance transmission and the different ways in which E. coli infects humans and animals. 

**Figure 3.**
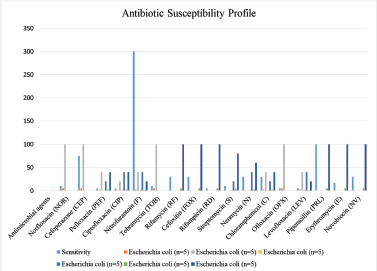
Antibiotic sensitivity report of *E. coli *isolated from examined fresh dromedary camel milk samples.

Figure 3 shows that E. coli isolates have the highest resistance to rifamycin (30 µg), cefoxitin (30 µg), streptomycin (10 µg), rifampicin (5 µg), erythromycin (15 µg), piperacillin (100 µg), and novobiocin (30 µg) and high susceptibility to norfloxacin (10 µg), cefoperazone (75 µg), tobramycin (10 µg), and ofloxacin (5 µg). Analysis of the antibiotic susceptibility of the isolated E. coli showed that all of them were multidrug-resistant as they showed resistance to more than three classes of antibiotics. 

It was found that 100% of the *E. coli* isolates tested were resistant to cefixime, levofloxacin (87.1%), piperacillin (78%), and nitrofurantoin (58%) [32]. Only 15% of the *E. coli* isolates tested were resistant to nitrofurantoin. There are many different types of antibiotics that *E. coli* can be resistant to, but the most common one is β-lactamase production. This gives the bacteria broad-spectrum resistance to cephalosporin and co-resistance to other types like aminoglycosides and tetracyclines [33].

Table 3 shows that the MAR ranged from 0.352 to 0.764 of the tested isolates (17 antibiotic agents). A calculated MAR > 0.2 indicated that the isolate came from a high-risk source of contamination and that there was abuse of antibiotics, while a calculated MAR < 0.2 indicated that this strain was identified from an area where antibiotics were used rarely or not used at all [25].

Multi-antimicrobial resistance in E. coli has become a perturbing topic that is threatening global public health. Improper choice of antibiotics, overuse, and consumption without prescription are causes of high antibiotic resistance in human and veterinary medicine worldwide, which leads to a high morbidity and mortality rate due to the low accessibility of effective antibiotics [34–36].

**Table 3.** Antibiotic sensitivity report and MAR index of *E. coli *strains from examined samples.

Organism/origin	Multidrug resistance MOs		Resistance pattern (*a*)	MAR index**
	No.	%		
***E. coli***				
Market milk	1	20	PEF, CIP, F, RF, FOX, RD, S, N, C, LEV, PRL, E, NV	0.764
Market milk	1	20	PEF, CIP, RF, FOX, RD, S, N, C, PRL, E, NV	0.647
Farm milk	1	20	RF, FOX, RD, S, N, PRL, E, NV	0.471
Farm milk	1	20	RF, FOX, RD, S, PRL, E, NV	0.412
Farm milk	1	20	RF, FOX, RD, PRL, E, NV	0.352

PEF, pefloxacin; CIP, ciprofloxacin; F, nitrofurantoin; RF, rifamycin; FOX, cefoxitin; RD, rifampicin; S, streptomycin; N, neomycin; C, chloramphenicol; LEV, levofloxacin; PRL, piperacillin; E, erythromycin; NV, novobiocin; TOB, tobramycin.

** MAR index = *a*/*b*, where *a* represents the sum of resisted antibiotics and *b* represents the total number of antibiotics used for sensitivity.

## Conclusion

Camel milk in Matrouh Governorate is consumed raw without processing, with a lack of refrigeration facilities in the desert during milking, handling, and transport until it reaches the consumers. To mitigate the risks posed by E. coli contamination of milk, good manufacturing practices must be followed. Additionally, an Egyptian standard must be established for raw camel milk.

## List of abbreviations

AHRI, Animal Health Research Institute; bp, base pair; BLAST, Basic Local Alignment Search Tool; EMB, Eosin–methylene blue; *eaeA*, intimin gene; h, hour; MAR, multiple antibiotic resistance; µg, milligram; ml, milliliter; *phoA*, phosphokinase; *stx*1, Shiga toxin-1; *stx*2, Shiga toxin-2; STEC, Shiga toxin *E. coli*; UPGMA, Unweighted pair group method with arithmetic mean; USA, United States of America.
